# Application of transport-based metric for continuous interpolation between cryo-EM density maps

**DOI:** 10.3934/math.2022059

**Published:** 2021-10-19

**Authors:** Arthur Ecoffet, Geoffrey Woollard, Artem Kushner, Frédéric Poitevin, Khanh Dao Duc

**Affiliations:** 1 Department of Mathematics, University of British Columbia, 1984 Mathematics Road, Vancouver, BC V6T1Z4, Canada; 2 Department of Computer Science, University of British Columbia, 2366 Main Mall #201, Vancouver, BC V6T1Z4, Canada; 3 SLAC National Accelerator Laboratory, 2575 Sand Hill Road, Menlo Park, CA 94025, USA; 4 Department of Zoology, University of British Columbia, 4200 - 6270 University Blvd., Vancouver, BC V6T1Z4, Canada

**Keywords:** optimal transport, interpolation, 3D shapes, cryo-EM, conformational heterogeneity, Primary 92C40, 65D05, Secondary 49Q10

## Abstract

Cryogenic electron microscopy (cryo-EM) has become widely used for the past few years in structural biology, to collect single images of macromolecules “frozen in time”. As this technique facilitates the identification of multiple conformational states adopted by the same molecule, a direct product of it is a set of 3D volumes, also called EM maps. To gain more insights on the possible mechanisms that govern transitions between different states, and hence the mode of action of a molecule, we recently introduced a bioinformatic tool that interpolates and generates morphing trajectories joining two given EM maps. This tool is based on recent advances made in optimal transport, that allow efficient evaluation of Wasserstein barycenters of 3D shapes. As the overall performance of the method depends on various key parameters, including the sensitivity of the regularization parameter, we performed various numerical experiments to demonstrate how MorphOT can be applied in different contexts and settings. Finally, we discuss current limitations and further potential connections between other optimal transport theories and the conformational heterogeneity problem inherent with cryo-EM data.

## 1. Introduction

For the past several years, advances in cryo-electron microscopy (cryo-EM) have led to a revolution in structural biology, for which the Nobel Prize in Chemistry was awarded in 2017. Mathematical and computational methods for the 3D reconstruction of proteins have played an important role in this revolution by allowing the technology to reach high resolution and stunning atomic-level detail [1]. Despite this spectacular success, the increasing use of cryo-EM on an ever wider range of biological systems continues to raise new challenges. Of these, one of the most pressing includes the modeling and inference of *conformational heterogeneity* [2, 3]: In practice, cryo-EM experiments yield 3D images, or so-called *3D density maps*, where each voxel captures the local Coulomb potential created by a molecule in 3D space [4]. Conformational heterogeneity in a given dataset has the potential to inform the functional mechanism of the molecule imaged —when this heterogeneity is interpreted as a discrete set of maps, the ability to simulate the motion between them would provide a key to generating relevant hypothesis about the mechanism and its biological implications.

A first approach to modeling conformational heterogeneity is to employ morphing-based techniques [5], with a standard method that consists of applying linear interpolation to obtain a trajectory of intermediate volumes between two given EM maps [6, 7]. However, these intermediate volumes are prone to generate unrealistic trajectories by blending the original density maps instead of displacing them. To mitigate these issues, we recently developed a software, called *MorphOT*, that alternately builds “displacement” interpolants, and yields continuous motion between EM maps [6]. The construction of these interpolants relies on the theory of optimal transport (OT) and recent advances that make tractable the computation of transport-based distances and barycenters of 3D shapes [8, 9].

While MorphOT was presented in [6] as a short note for cryo-EM practitioners that mainly focuses on the software, we provide in this paper a more extensive study of the method’s performance and applications. After briefly recapitulating the theoretical framework and our specific implementation for evaluating transport-based barycenters of EM maps, we assess how our MorphOT method compares with standard approaches on experimental EM maps, and detail the impact of the regularization parameter employed to approximate the OT distance, in terms of computational cost and accuracy. We further focus on two case studies of the spliceosome and the SARS-CoV-2 spike glycoprotein, to illustrate how our approach can be applied in the context of multiple maps to study conformational transitions, as well as some current limitations. Finally, we discuss the potential improvements and connections with other recent methods and problems in OT, that would help to fully study the extent of conformational heterogeneity from cryo-EM data.

## 2. Overview of the method and implementation for EM maps

### 2.1. Interpolation from regularized 2-Wasserstein distance

For two input 3D density maps *V*_0_ and *V*_1_, seen as continuous or discretized distributions mapping the Coulomb potential of a molecule in 3D space on $R^{3}$ (or in practice a finite subset, e.g. [−1; 1]^3^), finding a continuous *interpolation* consists of finding a trajectory *V*_*t*_ (0 < *t* < 1), joining *V*_0_ and *V*_1_ in the space of distributions. For a given metric (or distance function) assigned to the volume space, one can also define each interpolated distribution as a weighted barycenter between *V*_0_ and *V*_1_. For instance, *linear interpolants* $V_{t}^{\left(0\right)}=tV_{0}+(1-t)V_{1}$ are associated with the squared euclidean distance ∥.∥_2_, as

(2.1) $$V_{t}^{\left(0\right)}=\underset{V}{\text{argmin}}\left[t\|V_{0}-V{\|}_{2}^{2}+(1-t)\;\|V-V_{1}{\|}_{2}^{2}\right].$$

Alternately, one can consider performing a “displacement interpolation” [10, 11] based on the 2-Wasserstein distance ($W_{2}^{2}$), also known as the Earth mover’s distance (EMD). The EMD represents the minimal amount of work needed to move one mass density / probability distribution to another, with respect to a given cost function. To efficiently evaluate transport-based interpolants, Solomon *et al*. recently introduced OT-based optimization algorithms for large geometric domains [8], which we implemented and optimized for EM maps [6]. Briefly, the approach consists of regularizing the EMD with an entropy parameter *γ* > 0: for two initial and final distributions *μ*_0_ and *μ*_1_, defined on *X* and *Y* respectively, the entropy-regularized distance distance $W_{2,\gamma }^{2}(\mu_{0},\mu_{1})$ is given by

(2.2) $$W_{2,\gamma }^{2}(\mu_{0},\mu_{1})=\underset{\pi \in \Pi (\mu_{0},\mu_{1})}{\text{inf}}\left[\int_{X\times Y}d^{2}(x,y)\pi (x,y)dxdy-\gamma H(\pi )\right],$$

where *d*(·, ·) is the euclidean distance (in absence of any other information on the maps, it is chosen as the cost function), and *π* is called the *transportation plan*, which describes which amount of mass in x from *μ*_0_ gets sent to y in *μ*_1_. Π(*μ*_0_, *μ*_1_) is the collection of all measures on *X* × *Y*, with marginals *μ*_0_ on *X* and *μ*_1_ on *Y*, so the integral in Eq (2.2) yields the cost associated with all possible transport plans from *μ*_0_ to *μ*_1_ under the euclidean distance. *H*(*π*) is the entropy of the *transportation plan π*, defined as

(2.3) $$H(\pi )=-\int \int_{X\times Y}\pi (x,y)\;\text{ln}\;\pi (x,y)dxdy.$$

With $W_{2,\gamma }^{2}$, finding the interpolant of the EM maps *V*_0_ and *V*_1_ thus consists of solving the optimization problem

(2.4) $$V_{t}=\underset{V}{\text{argmin}}\left[\left(1-t)W_{2,\gamma }^{2}(\mu_{0},\mu_{1}{)}^{2}(V_{0},V)+tW_{2,\gamma }^{2}(\mu_{0},\mu_{1}{)}^{2}(V,V_{1}\right)\right].$$

The regularization makes the optimization problem *strictly convex*, ensuring the existence of a unique solution, which can also be seen as that of a *projection problem* with respect to the Kullback-Leibler divergence [12]. This projection problem can be resolved with an efficient algorithm using *iterated Bregman projections* [13] and *Lagrangian* optimization [8]. Furthermore, using a heat kernel makes the computation tractable in practice, by providing an alternative to storing the matrix of pairwise distance *d*^2^(*x*, *y*); *x*, $y\in R^{3}$ over a whole 3D grid of points [8], which would be prohibitive to implement for large 3D voxelized cryo-EM maps due to the matrix’s memory footprint. As is further shown in section 3 for EM maps, trajectories (*V*_*t*_)_0<*t*<1_ which are obtained from $W_{2,\gamma }^{2}$ tend to be more physically consistent and preserve shape integrity with more spread-out solutions being promoted as *γ* increases, in contrast with blended solutions produced by linear interpolation.

### 2.2. Implementation for EM maps

In practice, EM maps constitute voxelized 3D scalar fields, which represent a discretized form of the specimen’s Coulomb potential. Their box length typically varies from tens to hundreds of voxels, depending on the specimen’s physical size and the voxel size set by the microscopist during data collection (for example, a large macromolecule such as the ribosome, of diameter ~ 300Å, would necessitate a box size of 400 for a small voxel size of 0.75Å). Other factors to consider include the delocalization of information due to the point spread function / contrast transfer function, and padding used in typical discrete Fourier transform based signal processing workflows [14].

Although a Python library for OT already exists [15], it does not include convolutional Wasserstein distance computation, and is limited to 2D Wasserstein barycenters. For an efficient implementation applied to 3D grid voxels, we equivalently replaced the application of the heat kernel operator from the original algorithm in [8] by a convolution with a Gaussian of standard deviation *σ*^2^ = *γ*, which benefits from very efficient implementation in Python [16]. Using this optimized implementation of convolutional Wasserstein distance for 3-dimensional distributions (which to our knowledge is the first in Python), we developed a plug-in for the software *ChimeraX* that is commonly used to visualize EM maps [7]. The plug-in, called *MorphOT* [6], can run on both CPU and GPU, thanks to the enabling of GPU computing (with CUDA cores) in *ChimeraX*. While the GPU implementation is faster, having an option to run on the CPU may be desirable if GPU resources are being used by other processes on a workstation, or if a local machine used for map visualization lacks GPU resources, which is a common occurence in cryo-EM research groups. In addition to the method presented here, we also implemented a faster approximation for morphing trajectories, as a mixed solution that computes a fraction of OT barycenters and linearly interpolates between them, which can be useful in case of limited computational resources or high cost inherent with larger maps. Overall, our implementation of the present method thus allows the treatment of standard high resolution maps for computing barycenters and generating morphing trajectories, with good computational performance and improvement in accuracy over the standard current method, as detailed in the next section.

## 3. Performance study

### 3.1. Accuracy and comparison with linear interpolation

**Figure 1.**
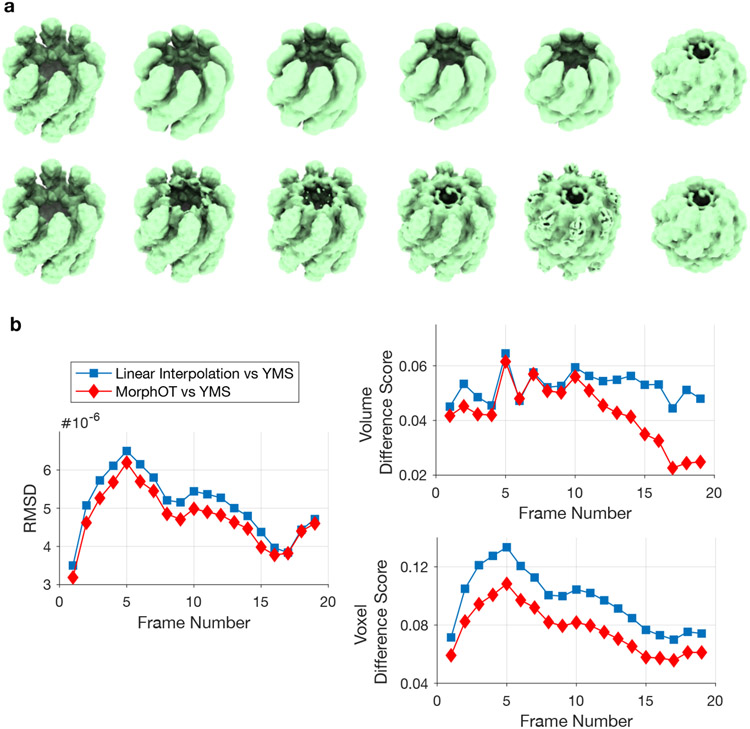
Comparison with linear interpolation: (**a**) Top: Interpolation obtained by running *MorphOT* with default parameters on two conformations of Mm-cpn [17] (EMDB 5137 and EMDB 5139). Bottom: Interpolation obtained with the linear method. (**b**): Using the Yale Morph Server Algorithm (YMSA) tool with default parameters in ChimeraX [18], we generated a structural morphing trajectory. We compared the resulting interpolants to the ones obtained by *MorphOT* (red diamond) and linear interpolation (blue square) using the RMSD (see Eq (3.1)), the global difference in volume (Eq (3.2)) and the voxel-wise differences (Eq (3.3)). Note that for frames 0 and 20 (start and end maps), not shown here, difference is always zero.

To evaluate the possible improvement of our transport-based interpolation over the linear interpolation (Eq (2.1)) implemented in *ChimeraX*, we first performed a quantitative comparison for two EM maps of Mm-cpn, an archaeal group II chaperonin [17], which were originally used to visualize trajectories produced by MorphOT [6]. These maps represent two states, closed and open, of the structure (see Figure 1) (a), so a realistic morphing trajectory should display a joint opening or closing of the branches forming the structure. As expected, transport-based interpolation produces gradual openings of each closed branch from the rings, showing a *displacement* of mass from one location to the other (Figure 1 (a), top row). In contrast, the linear interpolation makes rings from the open state already appear in intermediate states, with *teleportation of mass* occurring between closed and open conformations (Figure 1 (a), bottom row).

To confirm the visual impression that the transport-based interpolation yields more physically plausible transitions [6], we evaluated how the interpolations differ from one obtained by *structural morphing*. While OT and linear interpolations apply for electron density maps, *structural morphing* methods need a resolved atomic structure. Upon running the morph command in ChimeraX, we generated a structural morphing trajectory between the structures associated with the Mm-cpn maps. This structural morphing is based on a simple interpolation followed by energy minimization of each intermediate, which offers a compromise between chemical realism and computational efficiency [18]. While it does not in general guarantee that the interpolation is exact, the structures here are simple enough, so the motion obtained is at least physically plausible, with domains of the molecule moving while avoiding steric clashes.

To quantify how this structural morphing differs from linear and OT-morphings, we added a Gaussian distribution around each atom to generate EM maps for each frame. We then measured the difference with the linear and OT-morphings by first considering the classical root-mean-square-deviation (RMSD), defined for two maps *X* and *Y* as

(3.1) $$RMSD(X,Y)=\sqrt{\frac{\sum_{i=1}^{n}(X_{i}-Y_{i}{)}^{2}}{n}},$$

where *n* is the total number of voxels (= 421652 in our example), and *X*_*i*_, *Y_i_* are the map intensities in pixel *i*. As shown in Figure 1 (b), both OT and linear trajectories similarly differ from the structural (YMS) trajectory, with some variation across the frames, but with a smaller difference for the OT interpolation that reflects its better matching.

To interpret the improvement more precisely, we introduced two additional comparative measurements obtained after reducing the density maps into boolean maps, so each voxel gets assigned a value of 1 if the density is greater than a threshold value (e.g. 1% of the maximum density value), and 0 otherwise. For two EM maps *X* and *Y*, resulting in boolean maps $\tilde{X}$ and $\tilde{Y}$, we first define their difference in volume Δ_*Vol*_(*X*, *Y*) as

(3.2) $$\Delta_{Vol}(X,Y)=|Vol(X)-Vol(Y)|,$$

where $Vol(M)\;=\;\sum_{i=1}^{n}{\tilde{M}}_{i}$, and ${\tilde{M}}_{i}$ is the value of $\tilde{M}$ at voxel *i* (*M* = *X*, *Y*). As this measurement accounts for the global difference in volume (binarizing the maps allows to have a better sense of the effective occupied volume), it allows to compare how the interpolated maps capture the change in compactness between two input maps. After binarizing the data, the RMSD also yields a comparative measurement of the differences in voxel bits, that is tantamount to the shape-match score of similarity between maps used in Pintilie *et al*. [19]. We denote this difference in pairwise voxel intensity as

(3.3) $$\Delta_{Voxel}(X,Y)=\frac{1}{n}\sum_{i=1}^{n}\left[{\tilde{X}}_{i}(1-{\tilde{Y}}_{i})+{\tilde{Y}}_{i}(1-{\tilde{X}}_{i})\right].$$

According to these measurements, the OT trajectory is consistently closer to the YMS trajectory than the linear trajectory is, as shown in Figure 1. The difference in global volume is more significant in the second half of the interpolation, as the structure gets closer to the closed and more compact state (Figure 1 (b)). This reflects how the linear interpolation does not capture the increasing compactness of the structure, by keeping all the voxels in the open state that contribute to the barycenter, including those that are not in the closed state. In contrast, OT-interpolation turns off these same voxels as the mass in the open state gets displaced, reducing the difference in volume with the stuctural morphing. In the first half of the interpolation, linear and OT interpolations have similar global volumes, but our voxel-wise comparison, which removes the low-level contributions in the RMSD, shows that the OT-interpolation is consistently closer to the structural morphing across all frames (Figure 1 (b)). As linear interpolation does not continuously displace the mass of the branches and instead creates some in other parts of the grid as shown in Figure 1 (b), the newly formed mass leads to more non-overlapping voxels and local inconsistencies with the structural morphing, which are less observed with OT-interpolation. Interestingly, while our results suggest an improvement over the linear method, they also indicate some differences between the OT-trajectory and the structural morphing. We examine some potential explanations for these variations and how to improve the method in section 3.3 and the Discussion.

### 3.2. Computational performance

To illustrate the performance that can be achieved for different configurations, we benchmarked the time to interpolate between the same maps that we used in the previous comparison (Figure 1). As *MorphOT* can run on both CPU and GPU, with the CuPY Python package [20] as a backend, it thus offers various levels of performance for a wide range of machines and flexibility for the users, who can work at different resolutions and number of frames as needed. We report in Table 1 the times obtained for different configurations (using CPU or GPU) and grid size (from 60^3^ to 240^3^ voxels, which reflects the typical size of maps, as detailed in section 2.2).

The results indicate that the computation of a barycenter (time per frame) can be done in a range from 10^−2^s to up to few seconds for larger grid sizes with GPU (~ 10 to 10^2^) for CPU, which gives flexibility to quickly generate and visualize interpolating trajectories in practice. It is to be noted that while GPU and CPU implementations produced similar performance when MorphOT was first released [6], the recent port of Gaussian filter to CuPY’s array now allows our GPU implementation to take place almost exclusively in GPU memory, decreasing the interpolation time dramatically to ~ 10^−2^*s* per frame in a 60^3^ grid (see Table 1).

### 3.3. Impact of regularization on convergence and accuracy

**Figure 2.**
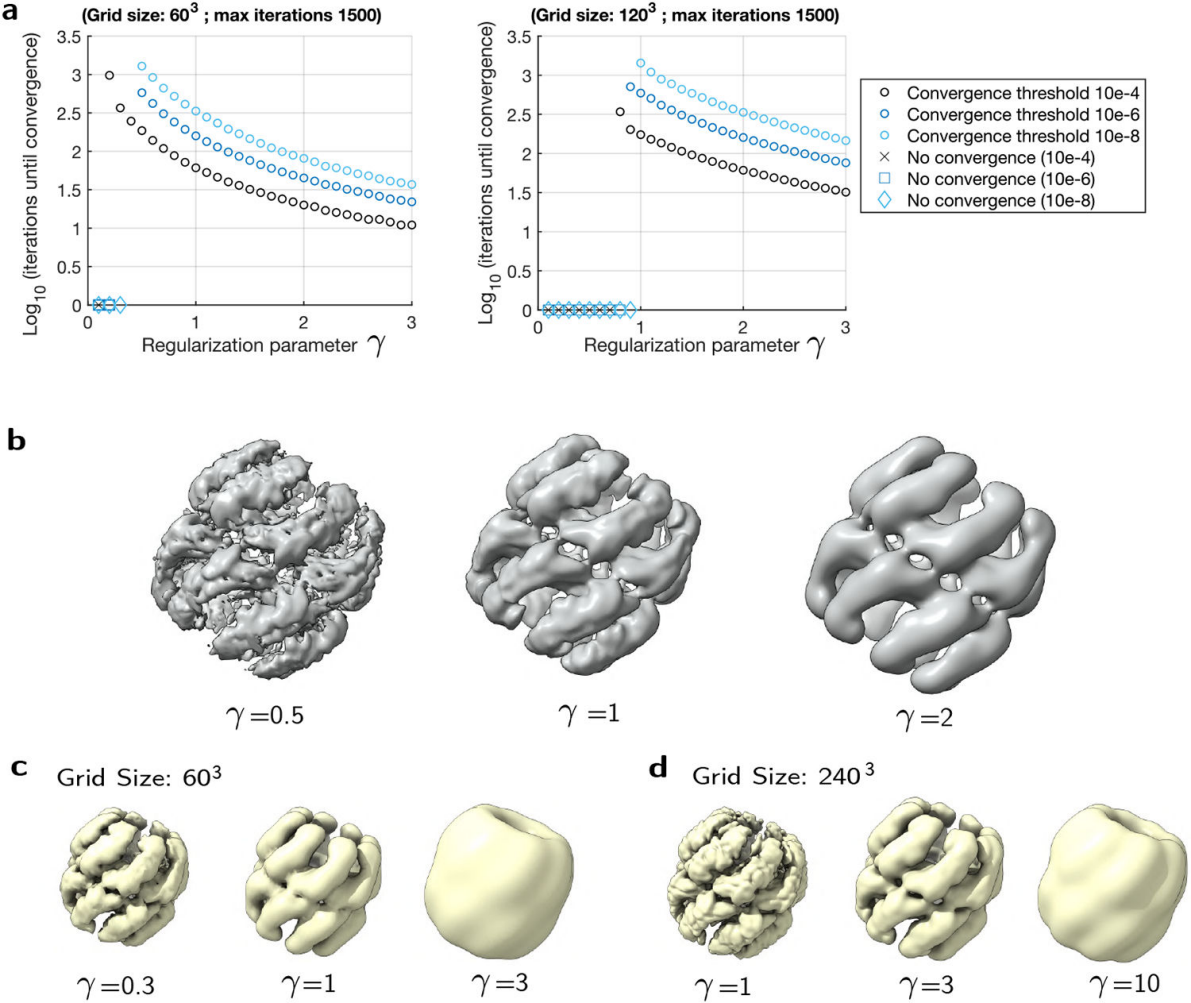
Impact of regularization on convergence and accuracy. We applied MorphOT to compute the isobarycenter of the same two maps as in Figure 1, for different values of *γ*. In **(a)**, we plot the number of iterations needed to reach different convergence thresholds (10^−4^, 10^−6^ and 10^−8^), defined by the *L*_2_ distance between the maps over two consecutive iterations, and for grid size 60^3^ (left) and 120^3^ (right). The maximum number of iterations was set at 1500, with “No convergence” shown when the threshold was not reached. **(b)**: We illustrate the impact of regularization on the sharpness of the interpolation, by displaying barycenters obtained for *γ* = 0.5, 1, 2 (with convergence threshold 10^−6^ and grid size 120^3^). The maps displayed are the ones obtained after 1500 iterations. **(c-d)**: Barycenters obtained from the same maps as Figure 2, but for different grid sizes (60^3^ and 240^3^) and values of *γ*.

Our current implementation relies on setting a parameter *γ* for the entropic-regularization of the EMD, which in practice blurs the optimal assignment as *γ* increases. On the other hand, the number of iterations to convergence to a solution scales exponentially when *γ* → 0 [21], which suggests that a compromise between accuracy and cost needs to be found to interpolate between EM maps. To better quantify this trade-off, we first studied how many steps the algorithm takes to converge for finding a barycenter, as a function of *γ*. To set our convergence criteria, we used difference threshold values (10^−4^, 10^−6^ and 10^−8^), and tested if the *L*_2_ distance between the maps over two consecutive iterations gets lower than this threshold (over 1500 iterations of the algorithm). We used the same maps as previously, and computed their isobarycenter (*t* = 0.5) with different grid size (60^3^/120^3^). The number of iterations required to converge decays supra exponentially as a function of *γ* (see Figure 2 (a)), with more iterations needed as the convergence criteria gets more stringent, or the grid size increases. At a grid size of 120^3^ and *γ* = 0.5, the threshold was not reached (with final maps shown in 2 (**b**)). Aside from convergence, the value of *γ* also affects the level of detail. We illustrate this in Figure 2 (b), which shows the interpolated maps at *γ* = 0.5, 1 and 2, with more regularization resulting in a loss of high resolution details. As the grid size increases, using the same value of *γ* also results in more high resolution details (see Figure 2 (c-d)), which however comes with a larger computational cost (Figure 2 (a)). Increasing the value of *γ* (e.g. from *γ* = 1 for grid size 60^3^ to *γ* = 3 for grid size 240^3^) allows to mitigate this cost, while keeping the same level of detail. On the other hand, at lower values of *γ*, the mass is more prone to scattering, resulting in a loss of integrity for the interpolated map (see Figure 2 (b) for *γ* = 0.5). In addition, numerical issues may appear as *γ* → 0, as the kernel operator becomes ill-conditioning with *γ* being too small with respect to the voxel size [8].

Currently the default parameters in the Chimera plugin of MorphOT are *γ* = 1, with a maximum of 1500 iterations, convergence threshold of 10^−9^, and downsampling to 60^3^ voxels. We recommend that the user adjusts *γ* such that they can achieve enough resolution in a reasonable time, depending on the map size and level of required details. To investigate higher resolution details, larger grid size should be used, with higher value of *γ*. These adjustments can be simply made on Chimera X (using the volume resample command) and MorphOT (with the reg option). In the next section, we illustrate how MorphOT can capture the conformational transitions of other biological systems.

## 4. Applications

### 4.1. Studying conformational transitions of the spliceosome

**Figure 3.**
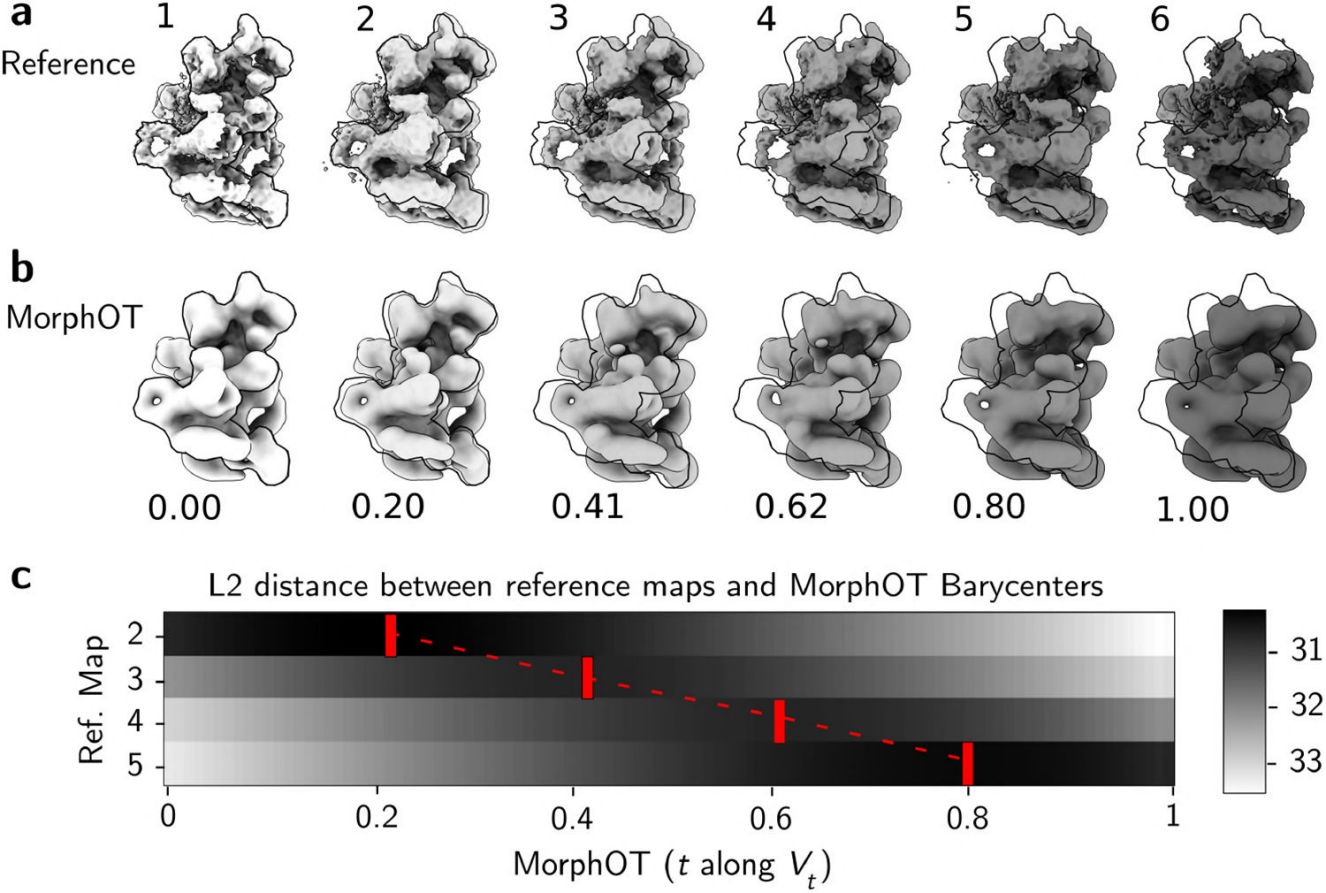
Inferring the sequence of conformations of the spliceosome with MorphOT (**a**): “Reference” ground truth trajectory from the first principal component (PC1) of heterogeneity as analyzed by [22], showing movement of the head region of the spliceosome. Maps 1-6 correspond to vol_000.mrc, vol_002.mrc, vol_004.mrc, vol_006.mrc, vol_008.mrc, vol_009.mrc along PC1 from [22] (publicly available at [24]). The contour of map 1 is outlined in black on other maps for orientation. (**b**): MorphOT barycenters matching the best with reference maps in (**a**), according to the RMSD. Interpolant index (*t* in Eq 2.4) is shown with each map (grid size 256^3^ pixels; *γ* = 3). The contour of the barycenter at *t* = 0.0 (reference map 1) is outlined in black on other maps. (**c**): Heatmap showing the *L*_2_ norm between each ground truth map barycenter (*t* = 0, 0.01, 0.02, …, 1.0). The red boxes show the closest barycenter to each reference map.

We first study the spliceosome, which has recently been used as a reference model for studying continuous conformational heterogeneity from cryo-EM [22, 23]. To get a reference trajectory, we used a family of maps that represents a low-dimensional latent space of conformations, obtained from single particle cryo-EM images [22]. We picked six reference maps that are uniformly distributed along the first principal component of heterogeneity in the latent space [22], as shown in Figure 3 (a). The associated trajectory keeps the foot and part of the body region of the spliceosome static, while the SF3b and helicase subcomplexes, approximately 1/4-1/3 of the spliceosome’s mass, are “smoothly transitioning from an elongated state to one compressed against the body of the spliceosome” [22].

We ran MorphOT to uniformly sample one hundred barycenters between maps 1 and 6, and studied the correspondence between these barycenters and the reference maps. In Figure 3 (b), we show the value *t* of the interpolant *V*_*t*_ that matches the best with each reference map, according to the RMSD (or equivalently the *L*_2_ norm), with the full heat map of pairwise *L*_2_ distances between reference maps and MorphOT barycenters in Figure 3 (c). These values conserve the global order of the reference maps, and correlate well with the coordinate in the latent space, as reference maps 2 to 5 (which respectively correspond to 20, 40, 60 and 80% of the segment joining maps 1 and 6 in the latent space) get assigned to *t* = 20, 41, 62 and 80 % for the barycenters, suggesting that the interpolation is consistent with the main heterogeneity component inferred from cryo-EM images.

While these results suggest that the distance scale and level of detail of MorphOT are suitable for this example of continuous heterogenity, we should also note that the published spliceosome trajectory used for reference here is generated from a learned latent space [22], which does not guarantee that it is physical. With various recent methods aiming to learn a low dimensional representations of conformational heterogeneity and expressing caution at physical correspondence [22, 25, 26], MorphOT provides a potential tool to assess how dynamical trajectories on the latent space differ from trajectories of Wasserstein barycenters, which have a simple physical interpretation of mass displacement.

### 4.2. Visualization of SARS-CoV-2 spike glycoprotein

**Figure 4.**
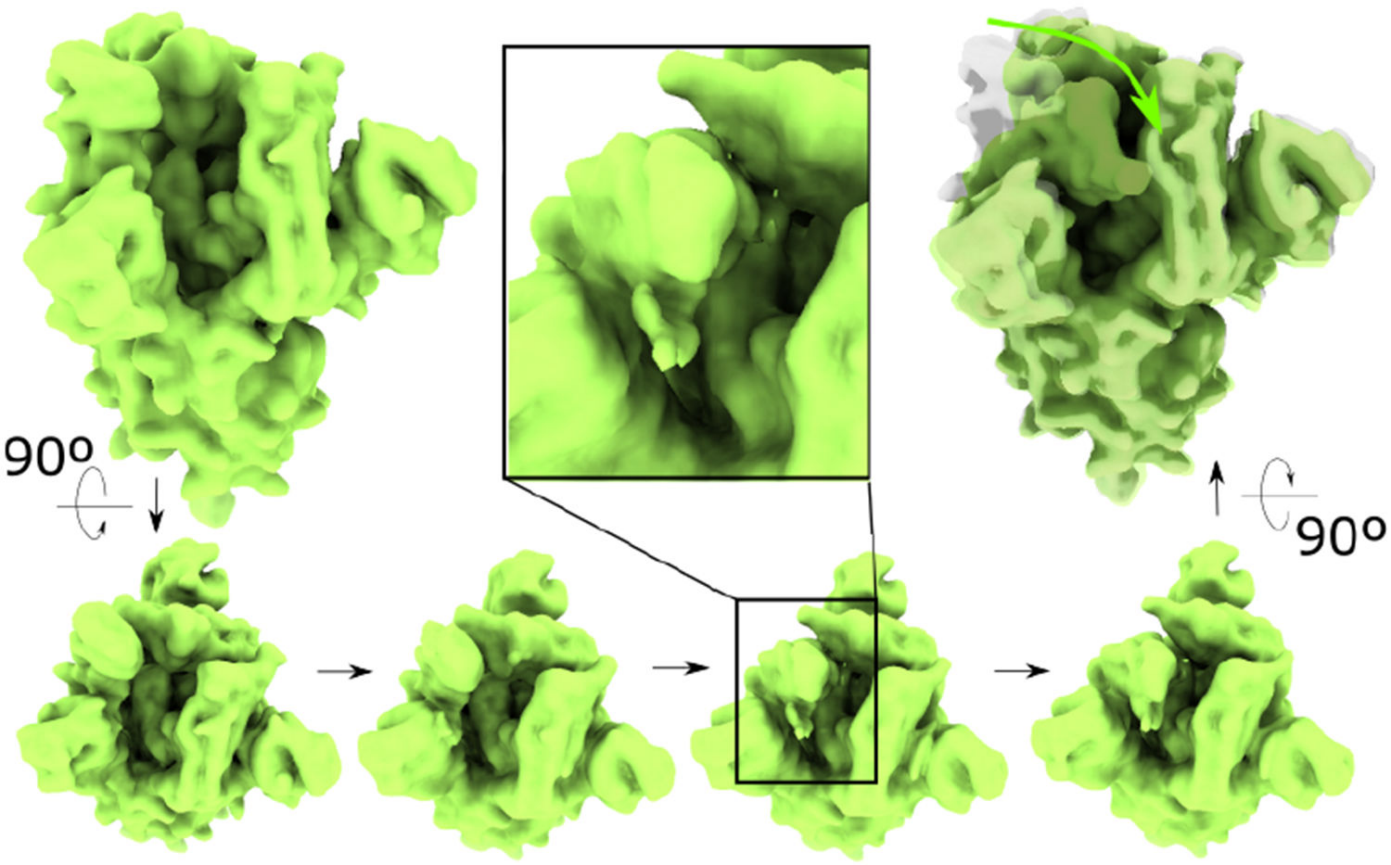
Example of MorphOT trajectory (with default parameters used) between two maps of the SARS-CoV-2 spike glycoprotein [27], shown at the top left and top right. Four snapshots along the trajectory are shown in the bottom of the image. **(center-top inset)** Detail of how the mass fragments while being transported.

We finally illustrate some limitations and directions for future improvement of the method, by testing MorphOT with recent maps of the SARS-CoV-2 spike glycoprotein [27]. The glycoprotein presents two states, which differ mainly by the rotation of a domain moving from the periphery of the protein to its core while the rest of the protein remains mostly unchanged. The morphing trajectory obtained shows a gradual motion of the domain instead of teleportation, as expected (Figure 4). However, we observe a transient loss of connectivity of the mass as it proceeds through the trajectory, with mass elements fragmenting away from the domain to join other parts of the molecule (see inset in Figure 4).

While this behavior is not unexpected from the point of view of OT theory (with amounts of mass moving along straight lines according to the transport map), it also highlights the fact that the transportation plan is not aware of some intrinsic properties of the molecule underlying the maps, such as its topology or connectivity. This example highlights the need to incorporate prior information in the transportation plan, in order to achieve morphing trajectories that respect the physics of the imaged object, either by adding constraints coming from experimental data or from atomic models.

## 5. Discussion

Using the Wasserstein distance as a metric for 3D volumes, we provided a new way to interpolate trajectories between different cryo-EM maps, which can be applied to visualize and gain more insights on the transitions occurring between different conformations of biomolecules. This method overcomes the limitations of the current standard linear method and has been implemented with the software *ChimeraX* [6], allowing structural biologists to easily apply it to new experimental data, or revisit previous studies. While introducing an OT-based method marked here a significant improvement for interpolation, and could more generally serve in the study of conformational heterogeneity [28, 29], we finally stress some limitations and lines of future work, which would further connect this fundamental problem in biochemistry with recent mathematical works and problems posed in OT.

1. **Scaling algorithm for entropy-regularized barycenter computation:** As we do not provide any fine-tuning of *γ* other than a heuristic based on this trade-off between computational efficiency and precision, a future improvement is to produce a refined scheme that can appropriately scale *γ* at each iteration. In this regard, a coarse-to-fine scheme was generally proposed for any type of cost function [21], which would be interesting to adapt in the context of large 3D maps. It would also be useful to quantify the relationship between *γ* and the grid size, and in particular, determine how to adjust *γ* if a map is initially downsampled for fast computation, while maintaining a visually similar barycenter.
2. **Modelling more complex transport-based trajectories:** An important point to stress is that although this transport-based metric allows to provide paths which are more physically sound, there is no guarantee that they are the true ones. In practice, Wasserstein barycenters displace the mass from the initial distribution along transport lines given by the tranport map [30], as illustrated in Figure 4. While we used the squared Euclidean distance as our cost function, one line of future work will be to produce trajectories associated with cost functions that are more realistic, and/or use prior information based on some biophysical properties of the system. Similarly to the so-called Multi-body refinement [31], which treats the heterogeneity in EM images as the result of the relative motion of few main rigid bodies, one can for example divide maps into rigid regions with distinct transport cost. In the context of OT, further constraints to insure incompressibility of the structure, stochasticity of trajectories, and other potential cost functions have been studied [9], and can also be adapted in our case.
3. **Extension to unbalanced optimal transport for variable mass:** When the two maps differ in composition and thus present different masses, the framework of the OT problem, which assumes input measures with identical mass, is not adapted. This suggests to extend the present approach within the framework of the so-called *unbalanced* optimal transport problem (UOT) [32]. In this context, OT algorithms that can handle mass variation have been proposed, and extend the existing fast OT methods (with regularization) to these new unbalanced problems [32, 33].
4. **Studying the inverse problem and learning the conformational energy landscape:** Ultimately, understanding the mechanisms explaining the dynamics between different conformational states can be cast as an inverse problem, aiming to recover the inner cost function associated with observed trajectories between two shapes/conformations. This inverse problem has been an active subject of research for the past few years, with some hypotheses made on the form of the cost function [34-36], and various methods, from deep learning [37] to Bayesian MCMC methods [38]. Those methods have in common that they heavily rely on the computation of many forward OT solutions, to learn the cost function. In this regard, recent neural network approaches [39] provide a promising and tractable approach to solve this inverse problem.

## Figures and Tables

**Table 1.** Benchmarking of transport-based interpolation. Using *MorphOT* and the *volume morph* command [6] with default values, we recorded the time to interpolate between the two EM maps shown in Figure 1. Each interpolation consists of 25 frames between the source and the end conformation. *GPU* and *CPU* testing were done on an NVIDIA RTX 2070 Super and a 3.5-GHz AMD Ryzen Threadripper 2950X respectively.

Gridsize	Walltime (s)		Time Per Frame (s)	
	*GPU*	*CPU*	*GPU*	*CPU*
240^3^	59.1	6876.1	2.40	275
120^3^	7.0	2304.6	0.30	92
60^3^	0.7	296.1	0.03	12
